# Evaluation of Oral Pregabalin as a Preemptive Adjuvant for Postoperative Pain in Patients Undergoing Coronary Artery Bypass Grafting With General Anesthesia and High Thoracic Epidural: A Randomized Controlled Study

**DOI:** 10.7759/cureus.70142

**Published:** 2024-09-25

**Authors:** Vipul Sharma, Chandipriya Singh

**Affiliations:** 1 Anesthesiology, Dr. D. Y. Patil Medical College, Hospital & Research Centre, Dr. D. Y. Patil Vidyapeeth, Pune, IND

**Keywords:** postoperative pain, pregabalin, randomized controlled trial (rct), sedation score, vas score

## Abstract

Aim

This study aimed to evaluate the effectiveness of oral pregabalin as a preventive supplement in managing postoperative pain in patients undergoing coronary artery bypass grafting (CABG) with a combination of general anesthesia and high thoracic epidural anesthesia.

Material and methods

This 18-month randomized controlled study at a tertiary hospital’s anesthesiology department included 62 American Society of Anesthesiologists (ASA) II or III patients aged 35-75 with left ventricular ejection fraction >35%. Placebo (Group B, n = 31) or pregabalin (Group A, n = 31) was randomly allocated. Group A got 150 mg of pregabalin the night before surgery and 75 mg on postoperative days 0 and 1, whereas Group B received a placebo. Postoperative pain was evaluated using the Verbal Numerical Scale and Visual Analogue Scale, while sedation was assessed with the Ramsay Sedation Scale. Statistical analysis was performed using SPSS for Windows, Version 16.0 (Released 2007; SPSS Inc., Chicago).

Results

In this study, pregabalin significantly reduced postoperative pain on Day 0 and Day 1 (p < 0.001) compared to the placebo. The pregabalin group exhibited higher sedation scores on Day 0 (p < 0.001), but there were no significant differences on Day 1. Inotrope requirements were similar between both groups.

Conclusions

CABG patients’ postoperative pain was greatly reduced by pregabalin without impacting sedation or inotrope needs. These data imply that pregabalin was a useful supplementary analgesic for CABG patients’ multimodal pain treatment.

## Introduction

Coronary artery disease (CAD) is one of the primary causes of illness and death globally. The most widely accepted approach/treatment for patients with multivessel/advanced CAD is coronary artery bypass grafting (CABG) [1]. This procedure involves surgically creating alternate paths using either harvested veins or arteries to overcome blockages caused by atheromatous deposits in the coronary arteries. CABG aims to enhance cardiac function and relieve anginal symptoms by restoring blood flow to the ischemic myocardium [2]. There are two main types of CABG: on-pump and off-pump treatments. Both types of surgeries mandate sternotomy, which leads to significant postoperative discomfort and pain that necessitates effective care [3].

The pain experienced after CABG surgery is severe and complex, originating from several sources, including the internal organs, musculoskeletal system, and nerves [4]. This intense pain has the potential to progress into long-lasting agony, which may greatly impair one’s ability to function. Effective postoperative pain management is essential since it significantly influences patient comfort, recovery, and overall results. Research has emphasized the intensity of this pain, as shown by Lahtinen et al. (2006), who found that 49% of patients reported severe pain when at rest, 78% while coughing, and 62% while moving after surgery [5-9]. Furthermore, a significant proportion of patients (31%) reported persistent discomfort during movement even after a year after the surgical procedure [9,10].

Opioids are often administered to patients undergoing CABG surgery to alleviate postoperative pain, which may be particularly difficult to manage in the context of the cardiac surgical environment, where the complexities of managing hemodynamics, the risk of respiratory depression, and the need for careful monitoring of pain and sedation levels present unique challenges [11]. Nevertheless, opioids are accompanied by a multitude of adverse effects, such as respiratory impairment, diarrhea, nausea, and constipation, which may hinder the process of recovery [12]. There has always been a search for pain management methods, such as multimodal analgesia, regional anesthesia, or the use of non-opioid analgesics, that effectively reduce pain while causing fewer negative side effects [13].

Pregabalin, a compound similar to the neurotransmitter gamma-aminobutyric acid, has shown potential as a viable option for proactive pain control [14]. It works by blocking certain channels of calcium in the central nervous system, which decreases the production of excitatory neurotransmitters that are involved in transmitting pain signals [15]. Pregabalin’s analgesic, anxiolytic, and anticonvulsant qualities make it appropriate for controlling postoperative pain in several surgical contexts, such as orthopedic, gynecological, and abdominal procedures [16,17]. Nevertheless, there is a scarcity of information about the effectiveness of this treatment in cardiac surgery, namely in patients undergoing CABG.

This randomized controlled study seeks to assess the efficacy of oral pregabalin as a preemptive adjuvant in managing postoperative pain in patients undergoing CABG with both general anesthesia and high thoracic epidural anesthesia. While preexisting studies, such as the systematic review and meta-analysis, have explored the use of pregabalin in cardiac surgery, our study differs by focusing on its specific application by combining general anesthesia with high thoracic epidural anesthesia for CABG patients. This combination is less commonly addressed in the literature. Additionally, our study aims to gather detailed data on the effects of pregabalin on inotrope needs, sedation levels, and overall patient outcomes during heart surgery that were not thoroughly examined in previous studies. The rationale for this study is to determine whether pregabalin offers additional benefits in terms of decreased postoperative pain levels, reduced sedative needs, and enhanced hemodynamic stability in this specific anesthetic context compared to a placebo, providing further evidence for its use in complex cardiac surgical procedures.

## Materials and methods

Study design

This randomized, double-blind, placebo-controlled trial was conducted at a tertiary-level hospital. Due to a good flow of patients, the study was completed within 12 months (May 10, 2023 to May 10, 2024), including patient recruitment, data collection, and follow-up. Patient recruitment was stopped once the sample size was achieved. The study was approved by the Institutional Ethics Sub-Committee of Dr. DY Patil Medical College, Hospital and Research Centre (ethics committee number: IESC/PGS/2022/145) and registered with the Clinical Trials Registry-India (CTRI number: CTRI/2023/05/052308). The study adhered to the ethical principles of the Declaration of Helsinki.

Randomization, allocation, and blinding

Randomization was performed using a computer-generated random sequence. Allocation concealment was ensured using opaque, sealed envelopes, which were opened sequentially only after patient consent. Blinding was ensured for both patients and investigators. One investigator administered the drug, while another, blinded to the intervention, recorded the outcomes in the operating theater. The placebo and pregabalin capsules were identical in appearance, ensuring that neither the patients nor the investigators knew the group assignments.

All participants who were randomized adhered fully to the assigned intervention and completed the study as per protocol. Therefore, a per-protocol analysis was conducted, which included only those patients who strictly followed the assigned intervention. This approach allows us to assess the efficacy of the intervention under ideal conditions.

Sample size

A total of 62 patients aged 35-75 years, classified as American Society of Anesthesiologists (ASA) II or III with left ventricular ejection fraction (LVEF) >35% and scheduled for elective CABG, were included in the study. Exclusion criteria included pregnancy, chronic opioid use, psychiatric illness, or known allergy to pregabalin. The sample size was calculated based on a power analysis designed to detect a clinically significant difference in postoperative pain scores between the pregabalin and placebo groups. Based on a power analysis to detect a medium to large effect size (Cohen’s d = 0.75) with a significance level (alpha) of 0.05 and a power of 0.8, the required sample size was calculated to be approximately 29 patients per group. To account for potential dropouts and ensure sufficient power, a total of 62 patients were randomized. The sample size was calculated using WinPepi Version 18.

Intervention

Participants were randomly assigned to one of two groups for this study. Group A, referred to as the Pregabalin group, included 31 participants who were administered 150 mg of pregabalin orally the night before surgery, followed by 75 mg on the night of surgery and 75 mg on the night of post-surgery day 1 (a total of three days). In contrast, Group B, the Placebo group, also comprising 31 participants, received a placebo at the same time points as Group A.

Preoperative preparation and evaluation

NBM status (more than six hours) and withholding of heparin, aspirin, and clopidogrel were confirmed according to the American Society of Regional Anesthesia and Pain Medicine guidelines [18]. Patients received Alprax 0.25 mg the night before surgery. After obtaining consent, a thorough preanesthetic evaluation, including history-taking and clinical examination, was conducted. Weight, pulse rate, blood pressure (BP), respiratory rate, and relevant clinical signs were recorded. Basic investigations (hemoglobin, urine routine, and microscopy) and special investigations (blood sugar, ECG, and chest X-ray) were performed as necessary.

Anesthetic technique

An 18-gauge epidural needle was inserted at the T4-T5 intervertebral space using the loss of resistance method, and an epidural catheter was placed 3 cm inside the epidural space with the patient in a sitting position during the preoperative period. Continuous monitoring was conducted during the epidural placement, including standard vital signs such as heart rate (HR), BP, and oxygen saturation. The patient was also monitored for signs of neurological compromise, and the procedure was performed under sterile conditions to minimize the risk of infection. A bolus of 10 ml 0.5% bupivacaine with 3 mg morphine was administered in 2 ml aliquots over 30 minutes, timed to coincide with the establishment of invasive pressure monitoring lines, including arterial pressure monitoring via a radial or femoral arterial line and central venous pressure monitoring via a central venous catheter. A 0.25% inj. bupivacaine infusion at 4-6 ml/hr was started preoperatively after the placement of the epidural catheter and establishment of invasive pressure monitoring. The infusion was continued throughout the surgery and into the postoperative period to provide continuous analgesia and maintain stable pain control. Premedication included inj. midazolam (0.02 mg/kg) and inj. fentanyl (2 mcg/kg). Induction involved inj. propofol (2 mg/kg) and inj. vecuronium (0.1 mg/kg) for muscle relaxation. Gentle laryngoscopy was performed to visualize the vocal cords, and an appropriate-sized endotracheal tube was inserted and fixed [19].

Intraoperative management

Intraoperative hypotension was managed with phenylephrine (50-100 mcg). One hour after epidural catheter insertion, heparin (4 mg/kg or 2 mg/kg) was administered, later reversed with protamine sulfate.

Postoperative care

**Figure 1:**
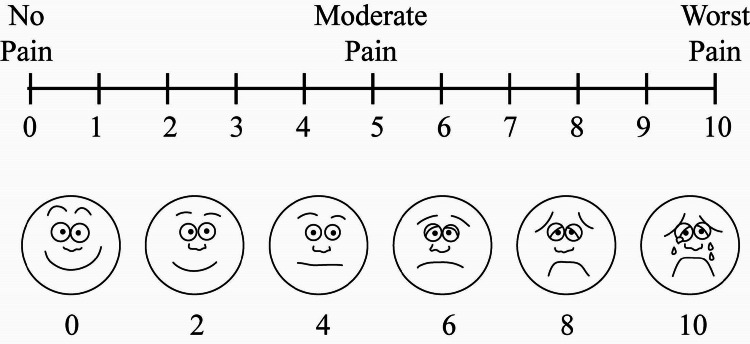
VAS for pain assessment VAS, Visual Analogue Scale

Patients were extubated in the ICU when hemodynamically stable, without ongoing blood loss, and inotropes were weaned off. The epidural was removed before starting antiplatelet therapy, preferably on POD 1. Breakthrough pain was managed with paracetamol (1 g) and diclofenac (75 mg). Postoperative monitoring included BP, HR, and saturation. Pain and sedation were assessed every two hours for 12 hours, then daily for three days using a Verbal Numerical Scale (0-10) and Visual Analogue Scale (VAS) scores (Figure 1).

Sedation and inotrope scoring

Sedation Score

The sedation scores are used to evaluate and monitor the level of sedation, and response levels are defined in Table 1, ranging from 1 to 6. This scale assesses varying degrees of responsiveness, with Level 1 indicating full wakefulness with agitation and Level 6 representing a state of deep sleep with no response to any stimuli [20].

**Table 1:** Sedation score levels and corresponding responses

Response	Level
Awake and agitated	1
Awake and calm	2
Drowsy but responsive to verbal commands	3
Drowsy but responsive to mild physical stimuli	4
Asleep but responsive to strong physical stimuli	5
Asleep with no response to stimuli	6

Inotrope Score (Vasoactive-Inotropic Score, VIS)

The calculation is obtained by summing the dopamine dosage, dobutamine dose, 100 times the epinephrine dose, 10 times the milrinone dose, 10,000 times the vasopressin dose, and 100 times the norepinephrine dose [21].

Statistical analysis

The data were assembled into an Excel spreadsheet created with Microsoft Excel (Microsoft Corporation, Redmond, WA, USA) and evaluated with SPSS for Windows, Version 16.0 (Released 2007; SPSS Inc., Chicago). Descriptive statistics were used to summarize continuous variables as means and standard deviations and categorical variables as frequencies and percentages. The chi-square test was applied to assess associations between categorical variables, with Fisher’s exact test used when expected cell counts were less than 5. A p-value of less than 0.05 was considered statistically significant.

## Results

**Figure 2:**
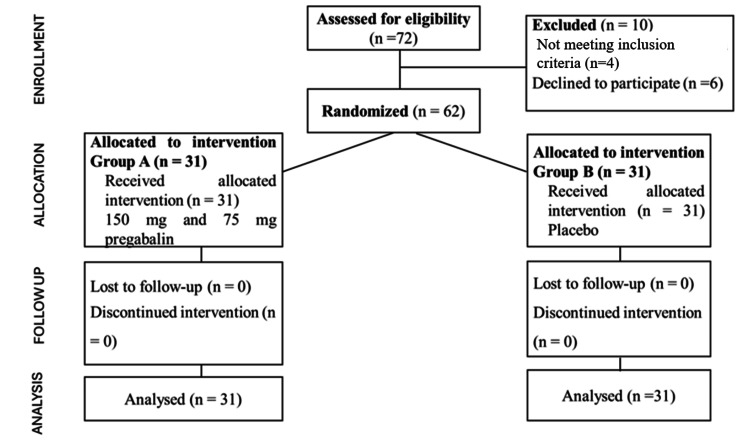
CONSORT diagram CONSORT, Consolidated Standards of Reporting Trials

The trial had 62 patients (Figure 2), with an equal distribution of participants between the pregabalin group and the placebo group (Groups A and B). The baseline parameters, such as age, gender, and ASA categorization, were similar among the groups. At predetermined intervals, the evaluated pain ratings, sedation levels, and the number of inotropes administered were assessed. The study revealed significant disparities in postoperative pain (p < 0.001) and sedation ratings (p < 0.001), suggesting that pregabalin may be effective in controlling postoperative pain in patients undergoing CABG.

The gender distribution showed a predominance of males in both groups, with 25 males (80%) in the Pregabalin group and 22 males (70%) in the Placebo group. Females made up six (20%) in the Pregabalin group and nine (30%) in the Placebo group, with each group consisting of 31 patients. The majority of participants in both groups were within the 51-70 age range, with the 61-70 age group being the most prevalent. Fewer individuals were represented in the younger (≤40 years) and older (≥81 years) age groups.

**Figure 3:**
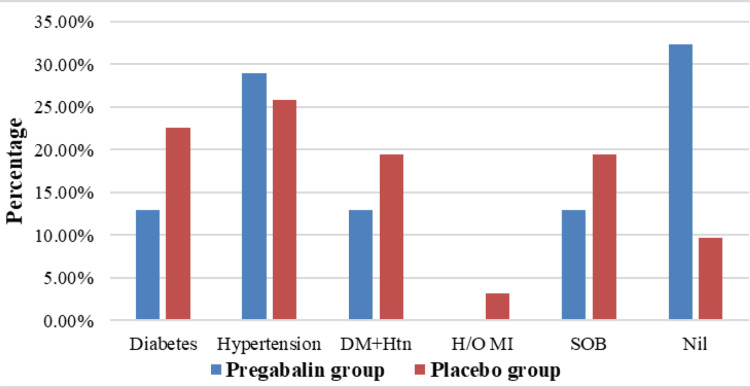
Prevalence of comorbidities in Pregabalin group and Placebo group patients DM, diabetes mellitus; DM+Htn, diabetes mellitus and hypertension; H/O MI, history of myocardial infarction; Htn, hypertension; SOB, shortness of breath

Figure 3 highlights the prevalence of comorbidities, with hypertension being the most common in both groups. Diabetes and diabetes with hypertension also showed notable prevalence. Shortness of breath was more common in the Placebo group, whereas a significant portion of the Pregabalin group reported no comorbidities. The least common condition in both groups was a history of myocardial infarctions.

Table 2 presents the blood investigations and echocardiographic (ECHO) findings between the study groups. The blood parameters, such as total leucocyte count, hemoglobin, international normalized ratio (INR), prothrombin time (PT), platelet count, and PT/INR ratio, did not exhibit any notable disparities between Group A and Group B (p > 0.05), suggesting comparable hematologic profiles. The ECHO results for LVEF were similar in both groups (p = 0.303), with similar percentages of patients in both groups having LVEF values below 40%. Nevertheless, notable disparities were seen in the ECG results. Group A exhibited a notable decrease in the number of patients with ST elevation (9.7% compared to 51.6%, p = 0.001) and an increase in the number of patients with T-wave inversion (48.4% compared to 22.6%). Group A also had a higher proportion of patients with normal ECGs (41.9% vs. 25.8%). These results suggested that while hematologic and ECHO profiles were similar, the ECG findings indicated a significant variation in underlying cardiac conditions between the groups.

**Table 2:** Blood investigations and ECHO findings among study groups Statistical tests: t-test and chi-square test; significant p-value <0.05 ECHO, echocardiography; INR, international normalized ratio; PT, prothrombin time; ST elevation, segment elevation; TLC, total leucocyte count

Parameters		Group A (n = 31)	Group B (n = 31)	p-value
Blood investigations (mean ± SD)	Hemoglobin	12.94 ± 2.4	13.13 ± 2.3	0.743
	TLC	6,863.9 ± 2,303.9	6,412.3 ± 2,275.6	0.441
	Platelet count	2.48 ± 0.6	2.45 ± 0.6	0.84
	PT	12.4 ± 1.3	12.5 ± 1.2	0.774
	INR	1.05 ± 0.11	1.06 ± 0.1	0.574
	PT/INR	11.9 ± 0.33	11.8 ± 0.59	0.515
ECHO (n, %)	<40%	5 (16.1%)	7 (22.6%)	0.303
	40-50%	13 (41.9%)	6 (19.4%)	
	50-60%	13 (41.9%)	18 (58%)	
ECG (n, %)	ST elevation	3 (9.7%)	16 (51.6%)	0.001
	T-wave inversion	15 (48.4%)	7 (22.6%)	
	Normal	13 (41.9%)	8 (25.8%)	

The comparison of HR, systolic BP (SBP), and diastolic BP (DBP) between two groups at various intervals is shown in Table 3. There were no statistically significant variations in HR between Group A and Group B at any time point, including baseline, intraoperative, and postoperative periods spanning from 15 minutes to two hours (p > 0.05). There were no notable variations in SBP across the groups before and after the surgery. However, over the postoperative periods, there was a little tendency toward lower SBP in Group B compared to Group A, but this difference was not statistically significant. Similarly, there were no statistically significant variations in DBP between the groups at the beginning and throughout the surgery. During the time after the operation, there were no notable variations in DBP between the statistically significant groups. In general, the findings indicate that there were no significant differences in HR, SBP, or DBP between the two research groups at various periods. This suggests that both groups had similar stability in their blood circulation throughout the period surrounding the surgery.

**Table 3:** HR, SBP, and DBP distribution in two groups at different intervals Statistical tests: t-test and chi-square test; significant p-value <0.05 BP, blood pressure; DBP, diastolic blood pressure; HR, heart rate; SBP, systolic blood pressure

Parameters		Group A (n = 31)	Group B (n = 31)	p-value
HR (mean ± SD)	Baseline	73.84 ± 8.7	75.32 ± 8.5	0.499
	Intraoperative	74.8 ± 9.2	76.7 ± 8.6	0.402
	Postoperatively 15 minutes	73.2 ± 9.1	74.1 ± 8.4	0.697
	Postoperatively 30 minutes	63.9 ± 10.1	64.8 ± 11.4	0.751
	Postoperatively 45 minutes	65.2 ± 10.4	66.7 ± 11.01	0.58
	Postoperatively one hour	68.4 ± 10.7	69.7 ± 7.4	0.573
	Postoperatively two hours	71.9 ± 10.5	69.6 ± 9.3	0.346
SBP (mean ± SD)	Baseline	123.5 ± 12.9	117.8 ± 11.3	0.07
	Intraoperative	119.7 ± 16.2	117.5 ± 18.4	0.616
	Postoperatively 15 minutes	97.6 ± 11.2	98.6 ± 11.9	0.735
	Postoperatively 30 minutes	92.2 ± 16.4	88.9 ± 13.3	0.395
	Postoperatively 45 minutes	91.9 ± 13.2	89.9 ± 11.9	0.532
	Postoperatively one hour	94.7 ± 10.7	91.9 ± 8.6	0.266
	Postoperatively two hours	93.5 ± 14.2	89.2 ± 11.04	0.195
DBP (mean ± SD)	Baseline	75.7 ± 8.2	71.4 ± 7.7	0.39
	Intraoperative	77.2 ± 9.1	73.1 ± 11.2	0.122
	Postoperatively 15 minutes	63.8 ± 8.4	63.9 ± 7.8	0.938
	Postoperatively 30 minutes	61.1 ± 11.9	63.03 ± 9.5	0.483
	Postoperatively 45 minutes	61.2 ± 8.1	59.5 ± 10.9	0.485
	Postoperatively one hour	65.3 ± 10.1	63.5 ± 10.5	0.486
	Postoperatively two hours	66.6 ± 10.4	63 ± 10.1	0.178

Table 4 presents a comparison of sedation scores (Ramsay Sedation Scale), pain scores (VAS), and inotrope scores (VIS) in both study groups at various time points. On Day 0, Group B had lower sedation scores than Group A (p < 0.001), indicating higher postoperative sedation in Group A. Significant differences in pain scores were observed on both Day 0 and Day 1 (p < 0.001), with Group A experiencing lower pain levels. However, inotrope scores showed no significant differences between the groups on either day (p > 0.05). Overall, pregabalin (Group A) improved pain control postoperatively without affecting sedation levels or inotrope requirements.

**Table 4:** RSS, VAS, and VIS distribution in two groups Statistical tests: t-test and chi-square test; significant p-value <0.05 RSS, Ramsay Sedation Scale; VAS, Visual Analogue Scale; VIS, vasoactive-inotropic score

Parameters		Group A (n = 31)	Group B (n = 31)	p-value
RSS (mean ± SD)	Baseline	2.06 ± 0.68	2 ± 0.73	0.72
	Day 0	2.03 ± 0.66	1.32 ± 0.47	<0.001
	Day 1	1.73 ± 0.78	1.61 ± 0.67	0.521
VAS (mean ± SD)	Day 0	2.10 ± 0.870	3.06 ± 0.727	<0.001
	Day 1	1.23 ± 0.497	2.71 ± 0.864	<0.001
VIS (mean ± SD)	Day 0	24.81 ± 13.7	25.3 ± 13.02	0.887
	Day 1	17.77 ± 11.9	18.45 ± 11.9	0.823

## Discussion

Primary outcome: postoperative pain reduction

The primary outcome of our study was the reduction in postoperative pain scores following CABG with the administration of pregabalin. Our research demonstrated significant reductions in pain scores in the pregabalin group compared to the placebo group. These findings align with existing research, such as Mishriky et al. (2015), which reported decreased opioid consumption and improved pain scores with pregabalin in cardiac surgery patients [22]. Similarly, Rai et al. (2017) found a significant reduction in pain scores and opioid requirements in patients undergoing cardiac surgery with pregabalin administration, consistent with our results. This reduction in pain scores is particularly important in the context of cardiac surgery, where effective pain management can significantly influence patient recovery and outcomes [23].

Our findings are further supported by the meta-analysis conducted by Carley et al. (2021), which included multiple randomized controlled trials involving cardiac surgery patients and revealed a consistent reduction in postoperative pain scores with pregabalin administration [24]. However, it is important to note that other studies, such as Baos et al. (2020), did not observe significant differences in pain scores between pregabalin and placebo groups. These discrepancies could be attributed to variations in dosing regimens and pain assessment methods, underscoring the need for standardized protocols in future studies [25].

Secondary outcomes: sedation levels and hemodynamic stability

In addition to pain reduction, we also investigated sedation levels as a secondary outcome. Our study found that patients in the pregabalin group experienced increased sedation on Day 0 postoperatively, a finding consistent with the results of Clarke et al. (2015) and Nimmaanrat et al. (2023), who also reported higher sedation levels with pregabalin administration on the day of surgery [26,27]. This finding is clinically significant, as sedation levels can impact postoperative monitoring and care.

Furthermore, we evaluated the hemodynamic stability of patients receiving pregabalin, specifically looking at HR and BP. Our study did not find any significant differences in these parameters between the pregabalin and placebo groups, aligning with the research by Gupta et al. (2018), which also reported no notable changes in HR or BP with pregabalin administration [28]. This suggests that pregabalin does not adversely affect hemodynamic stability in the postoperative period, making it a safe option for pain management in this patient population.

Limitations of the study

While our study provides valuable insights into the efficacy of pregabalin in managing postoperative pain after CABG, it is important to acknowledge its limitations. The relatively small sample size of 62 patients may limit the generalizability of our findings. Additionally, the follow-up period was limited to the immediate postoperative days (Day 0 and Day 1), which may not capture long-term outcomes and complications. Furthermore, we excluded patients with certain comorbidities such as epilepsy, liver disease, renal disease, and chronic pain syndromes, limiting the applicability of our findings to these populations. Another limitation is related to the blinding process. Although efforts were made to blind both participants and healthcare providers, complete blinding may not have been possible, which could introduce bias into the results. Additionally, the use of epidurals and other pain medications alongside pregabalin could have influenced the outcomes. While we attempted to control for these factors, the interaction between pregabalin and other analgesics may have affected pain scores and sedation levels, making it difficult to isolate the effects of pregabalin alone.

Lastly, our assessment of sedation was only conducted up to Day 1 postoperatively, not considering potential changes in sedation levels beyond this period. These limitations should be taken into account when interpreting the results, and further research with larger sample sizes, longer follow-up periods, and more stringent control of confounding variables is warranted to confirm our findings.

## Conclusions

Our study demonstrated that pregabalin administration significantly improved postoperative pain control in patients undergoing CABG without impacting inotrope requirements. The baseline characteristics between the pregabalin and placebo groups were well matched, ensuring that observed differences in outcomes could be attributed to the intervention. These findings support the potential efficacy of pregabalin as an adjunctive analgesic in CABG patients, offering a valuable option for multimodal pain management strategies in this population. Further research is warranted to explore optimal dosing regimens and patient selection criteria to maximize pregabalin’s benefits in postoperative pain management following CABG.
